# A Pilot Study to Evaluate the Efficacy of Self-Attachment to Treat Chronic Anxiety and/or Depression in Iranian Women

**DOI:** 10.3390/ijerph19116376

**Published:** 2022-05-24

**Authors:** Abbas Edalat, Massoumeh Farsinezhad, Makhsoos Bokharaei, Fateme Judy

**Affiliations:** 1 Algorithmic Human Development, Department of Computing, Imperial College London, London SW7 2RH, UK; 2 Faculty of Psychology and Education, Tehran University, Tehran 14459 83861, Iran; m.farsinezhad@ut.ac.ir; 3 Faculty of Education and Psychology, Alzahra University, Tehran 19938 93973, Iran; m.bokharaei@student.alzahra.ac.ir; 4 Department of Psychology, Islamic Azad University of Mahallat, Mahallat 37819 58514, Iran; f.judy@iaumahallat.ac.ir

**Keywords:** attachment theory, affectional bond, attachment object, childhood self, adult self, re-parenting, affect regulation

## Abstract

The aim of this pilot study was to evaluate the efficacy of the new Self-Attachment Technique (SAT) in treating resistant anxiety and depression, lasting at least three years, among Iranian women from different social backgrounds. In this self-administrable and algorithmic intervention, the participant, using their childhood photos, imaginatively creates an affectional bond with their childhood self, vows to consistently support and lovingly re-raise this child to emotional well-being. We conducted a longitudinal study with repeated measurement to evaluate the efficacy of SAT using ANOVA. Thirty-eight women satisfying the inclusion and exclusion criteria were recruited from different parts of Tehran. To describe the SAT protocols, a total of eight one-to-one sessions were offered to the recruits, the first four were weekly while the last four were fortnightly. The participants were expected to practice the protocols for twenty minutes twice a day. Two questionnaires, GAD-7 and PHQ-9, were used to measure anxiety and depression levels before and after the intervention and in a three-month follow-up. Thirty women completed the course. The change in anxiety level between the pre-test and the post-test was significant at *p* < 0.001 with effect size 2.5. The change in anxiety between pre-test and follow-up test was also significant at *p* < 0.001 with effect size 3.5. The change in anxiety between the post-test and the follow-up was significant at *p* < 0.05 with effect size 0.6. For depression, the changes between pre-test and post-test as well as between pre-test and follow-up were significant at *p* < 0.001 with effect size 2.3 and 3.1 respectively.

## 1. Introduction

Anxiety and depression are debilitating conditions which, together with substance abuse, are considered globally to represent the most common psychological disorders; they are correlated with each other and are also common features of many severe psychological disorders [1]. Based on the findings of a comprehensive assessment of prevalence and incidence of psychological disorders conducted in 197 countries between 1990 and 2013, depression is the predominant mental health problem worldwide, followed by anxiety [2]. In another systematic and meta-analysis of data spanning over three decades (1980–2013), based on 174 surveys in 63 countries, women had higher rates of mood and anxiety disorders, whereas men had higher rates of substance abuse [3]. Due to their prevalence, anxiety and depression have been proposed as the two axes of a two-dimensional model of neurotic (non-psychotic) mental illness in which common diagnostic concepts are identified as points in the two-dimensional space [1].

According to the report of Lancet Commission on global mental health and sustainability [4], “common symptoms of mental distress such as anxiety or low mood are associated with more total disability at a population level than diagnostically defined mental disorders”. The report furthermore stresses that the burden of mental health relative to physical health has risen in all countries in the past decades and that “when it comes to mental health, all countries can be thought of as developing countries”.

It is therefore instructive to consider the problem in a European country where data has been available. In UK primary care clinics in the 1990s, between a third and a quarter of all patients suffered from mental illness, and approximately half of patients with non-psychotic mental illnesses suffered from an undiagnosed mental illness, most commonly depression or anxiety [5]. These individuals may not have met the diagnosis for a definite category of mental illness, but had symptoms of both anxiety and depression. In the UK, there has been a poor prognosis of anxiety and depression: one half of sufferers experience relapse and a quarter become chronic, with the index episode lasting for at least two years [6]. Most patients with chronic mental disorders suffer from depression and anxiety [7]. Unsatisfactory short-term outcomes are associated with initial severity of symptoms, significant concomitant physical illnesses, severe social problems and material circumstances, as well as genetic risk scores and personality factors [6].

Psychotherapy and pharmacological interventions are the two fundamental treatment methods available for mental disorders. A comprehensive meta-analysis of studies to compare the results of cognitive behavioural therapy (CBT) and pharmacological interventions has established that these two treatment methods have largely the same level of efficacy; however, a moderator analysis showed that participants in anxiety studies that included comorbid depression were more likely to benefit from pharmacotherapy, whereas participants in anxiety studies that excluded depression were more likely to benefit from CBT [8]. A meta-analysis of a large number of studies comparing the preferences of psychiatric patients for psychotherapeutic interventions versus pharmacological interventions has concluded that 75% of patients prefer psychotherapy; and a sensitivity analysis shows that younger patients and women are significantly more likely to choose psychological treatments [9].

Patient preference for psychotherapy has driven research investigating new and more effective psychotherapeutic interventions, specifically for chronic and resistant conditions. In the research literature, chronic depression is differentiated from non-chronic major depressive disorder (MDD) on many clinical, psychosocial, and familial variables, since it is usually associated with higher comorbidity, functional impairment, suicidality, personality disturbance, childhood adversity/maltreatment, and familial liability for mood disorders; see [10]. In the Diagnostic and Statistical Manual of Mental Disorders (DSM-V), persistent depressive disorder (dysthymia) (PDD) is introduced as “a consolidation of DSM-IV-defined chronic major depressive disorder and dysthymic disorder” [11]. DSM-IV also categorises Generalized Anxiety Disorder (GAD) in a person who reports chronic, excessive worry which is not related to particular circumstances, and symptoms of physiological arousal such as restlessness, insomnia, and muscle tension [11].

In recent decades, an increasing number of psychological interventions—in particular derivatives of CBT including group CBT and mindfulness-based CBT, but also several other therapies such as interpersonal therapy (IPT), dialectical behavioural therapy (DBT), schema-focused therapy (SFT), compassion focused therapy (CFT), mentalisation based therapy (MDT), structured clinical management, and acceptance and commitment therapy (ACT)—have been introduced to tackle such chronic conditions with a number of Randomised Clinical Trials measuring the efficacy of these treatments [12,13,14,15,16,17].

These therapies aim to identify and redress cognitive defects, as in classical CBT, or to regulate emotions, as in CFT or ACT. They are generally focused on the ‘here and now’. In contrast to psychodynamic or psychoanalytical interventions, they do not generally examine the childhood environment and experiences, with the notable exception of SFT, which is highly influenced by John Bowlby’s attachment theory and has been shown to be effective in tackling chronic conditions such as personality disorders [14].

### 1.1. Attachment Theory

Attachment theory was introduced by John Bowlby in the 1960s and 1970s [18,19,20] and has since developed into a main paradigm in developmental psychology with wide impact in many related areas including psychotherapy.

The basic tenets of attachment theory are supported by a large number of studies over many decades [21]. A wide-ranging secondary analysis on attachment insecurities, based on hundreds of cross-sectional, longitudinal, and prospective studies of both clinical and non-clinical populations, concluded: “[A]ttachment insecurity was common among people with a wide variety of mental disorders, ranging from mild distress to severe personality disorders and even schizophrenia” and that “attachment insecurity is a major contributor to mental disorders” [22,23]. In addition to attachment insecurity, neglect and abuse by primary attachment figures become the source of great distress, insecurity and instability for children who then question the trustworthiness of their caregiver. These children often continue to experience repeated trauma and thus acquire the expectation that these episodes do recur, resulting in hypervigilance and chronic anxiety and leading to the maldevelopment of a brain “for survival”, without acquiring the capacity for self-regulation of emotions [24,25,26].

On the other hand, several experiments—using a technique called ‘security priming’—have been able to artificially activate mental representations of supportive attachment figures and thereby improve the mental health of individuals suffering from various mental disorders [22].

John Bowlby himself believed that attachment continues in one way or another later in life and can include attachment to abstract concepts rather than just to individuals: “Probably in all normal people [attachment] continues in one form or another throughout life and, although in many ways transformed, underlies many of our attachments to country, sovereign, or church” [27].

In fact, in the past few decades, the notion of a religion or a deity as an attachment object has been extensively examined in Christianity, Judaism and Islam by several groups of researchers [28,29,30,31].

### 1.2. Self-Attachment Technique

The self-attachment technique (SAT), as introduced in [32,33,34], is informed by John Bowlby’s attachment theory. It is based on establishing an affectional bond with one’s childhood self—subjectively experienced as falling in love with the child—and subsequently ‘re-parenting’ that child to emotional maturity by emulating the optimal parent-child interactions at the time of the child’s distress. This procedure, in practice, means learning to take care of oneself in a mature way; in SAT, this caring behaviour is conceived as the interaction of two actors within the individual, resembling a parent raising a child.

The individual’s mindset and behaviour are considered as the interaction of two agents, the childhood self and the adult self: The childhood self, mentally represented by the individual’s childhood photos, is conceived as the emotional self which is usually dominant when the individual is under stress. The adult self is conceived as the rational self which is usually dominant in the absence of stress. The adult self establishes first a compassionate connection with their childhood self, using their favourite childhood photos. Then, by reciting their favourite love and jolly song while looking at their favourite childhood photo and focusing on what they cherish and celebrate about their childhood, the adult self creates a passionate, imaginative bond with that child.

A vow is then made by the adult self to look after the child whenever they are in distress. This requires the adult taking up the challenge to comfort the child and moderate their arousal level whenever the individual is overwhelmed or affected by negative emotions. In practice, this comforting role is played by emulating the actions of a ‘good enough’ primary caregiver when their child is distressed [35]. It consists of projecting one’s negative emotions to one’s childhood photo, imaginatively embracing the child, loudly reassuring them, and giving oneself a head or neck self-massage (a close counterpart for cuddling a real child in distress). There are also several self-administrable protocols in SAT that mimic the actions of a primary caregiver, based on singing, dancing, laughing and playing, to enhance and maximize positive affects.

SAT thus represents an algorithm to re-run the emotional development of the individual while closely following the optimal parent-child interactions in real life. Since the affectional bond between the adult self and the childhood self is imaginative, SAT promotes a type of spirituality for emotional growth and well-being. To make a comparison with religion as an attachment object, in SAT the adult self rather than a deity becomes the attachment object for (the childhood self of) the individual.

The theoretical basis of the bond-making in SAT is the self-love of any individual which Freud considered a common component of human psyche and called primary narcissism [36]. Bowlby argued that separation anxiety is an injury to primary narcissism [18]. It is hypothesized in SAT that this affectional bond with the childhood self induces dopamine in the brain’s reward circuitry as is the case in romantic love [37,38], maternal love [39] and love of God [40].

Dopamine in the brain is thought to invoke response–reward and stimulus–reward associations for the control of motivated behaviour by past experience [41]. Based on this hypothesis, we can expect that by activating the reward circuitry of the brain, SAT can lead to more hope, energy and incentive in the individual for practicing its protocols to improve mental health. In this way, it is hypothesized that SAT can redress problems in the early attachment interaction of an infant with their primary caregivers in the preverbal period of life, when the parent maximises the positive affects in the infant by singing, dancing and play and minimises their negative affects by embracing, cuddling and affection [24,25].

SAT consists of a set of practical exercises that are learned in 8 to 12 weeks and are intended to be practised for 20 min twice a day in this period; the list of exercises can be found in [33]. It is hypothesized that repetition of these protocols, by long term potentiation and neuroplasticity, will in time create optimal and robust neural activation patterns that challenge the suboptimal circuits in the brain, resulting in more adaptive cognitive and behavioural prototype patterns corresponding to secure attachment. The exercises can eventually be integrated in the individual’s routine schedule by turning the adult self into an attachment object for the childhood self to earn secure attachment and emotion self-regulation.

SAT has been supported by various computational models [34]. These include the basic frameworks (i) and (ii) below for the overall concept of SAT, as well as the additional models (iii), (iv) and (v) that correspond respectively to the three stages of SAT, namely connecting compassionately with the childhood self, creating an affectional bond with the childhood self and practicing protocols to enhance positive affects and reduce negative affects, respectively.

(i) In [32,42,43], the Hopfield network, the first artificial neural model of associative memory, has been used to show how behavioural and cognitive prototypes can be modelled in artificial neural networks as strong patterns stored in the artificial brain. The dominant sub-optimal prototype can undergo a fundamental change by creating through repeated learning—corresponding to neuroplasticity and long-term potentiation—a new strong pattern that represents a more optimal behavioural and cognitive prototype. The overall process thus reflects the psychotherapeutic process.

(ii) In [44], the various styles of attachment in the relationship between a parent agent and a child agent have been modelled in the setting of active inference and one-player game theory. In the simplest model, the parent’s behaviour is governed by their probability of attending to the distressed child, whereas the child has one of three kinds of possible actions: seeking proximity, guarded seeking and avoidance, each with a specific pay-off for the child. The system has three equilibria which can be identified, respectively, as (1) avoidant attachment, (2) anxious attachment and (3) secure attachment, corresponding, respectively, to low, moderate and high probabilities of parental attendance. A more complex model for disorganised attachment has also been developed in the above paper. This framework provides a model of SAT in which the adult self, by gradually increasing the probability of attending to the needs of the childhood self throughout the intervention, turns avoidant and then anxious attachment of the childhood self to secure attachment. In case the parameter interval for anxious attachment is very short, the avoidant attachment can turn directly to secure attachment.

(iii) The compassionate attitude towards the childhood self in the first stage of SAT has been modelled based on a framework by Numan [45] in the neural circuits in the brain using available data on caregiving behaviour in human and animal studies [46]. A more detailed discussion of this model is provided in the Section 4 in relation to CFT.

(iv) In [47], a neural model of bonding circuitry based on the orbital prefrontal cortex (OFC) is considered, in which the OFC mediates between facilitative and stress reactivity to social stimuli, via the dorsomedial and paraventricular nucleus of the hypothalamus. By integrating more recent neuroscientific results, a computational model is developed and, using simulations of the model, it is postulated that introducing additional reward could drive a further mechanism: a counter-conditioning and re-balancing mechanism between activation of these networks, via increasing prefrontal-driven inhibition of the central nucleus of the amygdala. It is hypothesised in the above paper that such a process may be involved in self-bonding that takes place in SAT.

(v) In [34], the practice of SAT has been modelled in the brain by reinforcement learning (Q-learning), based on a neural model of pathways for emotional-cognitive decision making developed by Levine. This framework is then integrated with a competitive Hopfield network built from strong patterns for the six basic emotions and the core SAT protocols. A successful SAT intervention is then considered as the process that brings about change, induced by reinforcement learning, from strong patterns of negative emotions to strong patterns of positive emotion.

Another significant aspect of the new intervention is that SAT is ultimately a self-administrable and algorithmic technique that can be available to patients by technological tools such as virtual reality (VR) environments and chatbots, thus potentially reducing the need for client-therapist interactions and making the intervention scalable. We briefly describe these two technological tools that have so far been developed.

The interactive VR platform for SAT [48] features a virtual assistant and a customised child avatar that is created from the user’s favourite childhood photo. The virtual agent interacts with the user and based on an emotion recognition algorithm provides suggestions for the user to undertake an appropriate self-attachment sub-protocol. The user can also interact with the child avatar for example by embracing them.

The chatbot [49] is designed to coach the user in practicing the SAT protocols; it is a rule-based framework that is augmented with an AI-platform for engaging with the user in an empathetic, safe, fluent and non-repetitive way. The AI agent suggests SAT protocols in different contexts that are informed by the user’s current emotion and their past interactions with the protocols.

In this study, we investigated the research question of whether the self-attachment technique—based on eight individual face-to-face sessions with a therapist over 12 weeks and daily practice of SAT protocols twice a day for 20 min each—can alleviate resistant depression and anxiety in Iranian women. We measured levels of anxiety and depression, respectively, using GAD-7 and PHQ-9 questionnaires in pre-test, post-test and three-month follow-up test. A longitudinal in-group study was conducted and the results were obtained by ANOVA procedure.

## 2. Materials and Methods

### Research Design and Participants

This study was conducted before the Covid-19 pandemic. To recruit volunteers from a cross-section of the population, three introductory workshops on SAT were organised at Baharan Cultural Center (Tehran 17), Ferdows Cultural Center (Tehran 5) and Salman Farsi Cultural House (Tehran 17), and the study was also promoted in North Afsarieh Neighbourhood House (Tehran 15). The recruitment took place from the above three centres as well as three private clinics in west, central and east Tehran.

In [50,51], a sample size of at least 30 is recommended for a pilot in order to determine the sample size of a subsequent randomized control trial. We therefore aimed to recruit 30–40 volunteers for this study.

Inclusion criteria: women aged 20–50, suffering from chronic depression or anxiety, or both, for at least the past three years; and a minimum level of education (high school diploma) to be able to understand the concepts and write a report on their treatment exercises.

Exclusion criteria: psychotic illness, personality disorders, substance abuse, any other ongoing form of psychotherapy.

If the participants were on medication they were expected to continue taking it during the intervention without changing to any new medication.

The research was a quasi-experimental design performed within a group. Ethical approval for the study was granted by the Psychology and Counselling Organization of the Islamic Republic of Iran. In the selection process, volunteers were assessed using the GAD-7 anxiety questionnaire and the PHQ-9 depression questionnaire.

These questionnaires are widely used in primary care in the UK, and also in the US. They have been developed in the UK for Improving Access to Psychological Therapies (IAPT) services, which provide evidence-based psychological therapies to people with anxiety disorders and depression. Their reliability and validity have been shown in [52,53] respectively; see also [54]. GAD-7 has seven questions related to anxiety symptoms experienced in the previous two weeks with a Likert scale 0–3 and scores as follows: 0–4 minimal anxiety, 5–9 mild anxiety, 10–14 moderate anxiety, 15–21 severe anxiety. PHQ_9 has nine questions related to depressive symptoms experienced in the previous two weeks with Likert scale 0–3: Total scores of 5, 10, 15, and 20 represent cut-off points for mild, moderate, moderately severe and severe depression, respectively.

We note that, in comparison, Beck’s Depression inventory has 21 questions on the current symptoms of the individual, whereas Beck’s anxiety inventory, which has also 21 questions, measures the individual’s symptoms in the previous month. Thus, neither Beck’s inventories nor the IAPT questionnaires GAD-7 and PHQ-9 are sensitive enough to measure the long-term chronic aspects of anxiety or depression.

For our study, to check if the symptoms of depression and/or anxiety have endured for at least three years, a semi-structured interview was conducted by trained psychologists for at least one hour with each of the volunteers.

Fifty-two individuals applied to take part in the study. Fourteen of them did not meet at least one of the selection criteria. Thirty-eight volunteers, who were diagnosed with depression and/or anxiety lasting for at least the previous three years and who understood the concepts of self-attachment and accepted commitment to practice the protocols for 20 min twice daily, were selected for the study. They signed a consent form to undertake the daily practice of the protocols and briefly report their experiences in a diary.

The treatment protocol took place over eight 50-min-long one-to-one therapeutic sessions, which focused on explaining the SAT protocols and discussing the experiences the participants had gone through between sessions. The first four sessions were conducted on a weekly basis, and the last four were undertaken bi-weekly. Overall the treatment lasted for three months. In the first two weeks of the intervention, eight volunteers dropped out giving various reasons including objection by their husbands, pregnancy, family issues, physical illness, difficulty in practicing the protocols and the long distance they needed to travel for the face-to-face sessions. The data for these eight dropouts were not considered in the analysis of the study.

In the preliminary session, when the consent form was signed, each participant provided a list of significant childhood experiences together with their associated memories in relation to their primary caregivers and they were asked to bring two photos of their childhood, a favorite photo called, for convenience, “happy” and a least favorite photo called “unhappy” to the next session. Participants were also asked to imagine a derelict old house representing their inner world that they would be mending and renovating during the treatment. This notion of a house has some parallels with the ‘archetypal home’ representing the psyche in Jungian therapy [55]. At the end of the preliminary session, each participant was asked to choose a favorite jolly love song and bring the lyrics with them to the next session.

Three qualified psychotherapists who had previously trained in CBT and some other well-established interventions such as SFT conducted the intervention. They had previously also been trained in SAT in three different workshops in previous years and had practised SAT with some of their clients for at least two years. The self-attachment protocol in [33] was used to formulate sub-protocols for each of the eight sessions, which would be practised daily by each participant until the next session. The therapists were supervised by a psychiatrist and met on a regular basis to coordinate their work and ensure that the intervention in each session was uniform and fidelity to SAT was respected throughout the treatment. To ensure compliance and engagement of the participants with SAT, the first forty minutes of each session were dedicated to learning and discussing SAT sub-protocols. Any problems participants had in their everyday relationships could only be discussed with their therapist in the last ten minutes of the session.

Here is a summary of the eight intervention sessions:

Session I: The participant connects compassionately with their childhood self, using their two childhood photos. By looking at the “happy” and “unhappy” photos, they imagine their childhood self, experiencing both positive and negative emotions, while expressing compassion to the child in both cases.

Session II: The participant bonds with their childhood self by reciting their favorite jolly song. The participant is invited to imaginatively adopt the childhood self as their own child in the week ahead and be aware that loving, supporting and looking after the child is a task for life.

Session III: Two exercises for the “unhappy” child are practised each for 20 min: one for reprocessing negative childhood experiences and the other for reprocessing negative affects due to difficult current emotional problems. In both cases, the negative emotions are projected to the “unhappy” childhood photo and the adult self is tasked with comforting the child by loud verbal assurance and cuddling them which is imaginatively practised by the participant using a head and face self-massage.

Session IV: The experience of practicing the exercise of the third session is discussed and the difficult parts of the exercises are practised in the session.

Session V: As in the previous session, the experience of practicing the exercise of the third session is discussed and the difficulties encountered in the past two weeks are addressed and the harder parts are practised in the session. Then, the exercise for overcoming negative affects and earning secure attachment using the renovation of the house is undertaken.

Session VI. The exercise in the previous session is practised. Then the sub-protocol for socialising the childhood self is worked through. In this way, the participant learns to contain any strong anti-social tendencies such as hate and revenge.

Session VII. First the sub-protocol for socialising the child is repeated and then the exercise related to developing a new internal working model is practised. This is based on earning secure attachment in the renovated house.

Session VIII: All the problematic aspects of the SAT protocol are reconsidered and the need to practice the exercises after the treatment in order to retain the new habits is emphasised.

Among the thirty participants there were several women who had previously undertaken some well-established therapies but had not significantly benefitted from them. There were also two women who had experienced sexual abuse as children and there were two others who had traits of Obsessive Compulsive Disorder (OCD).

After the intervention, the participants’ levels of anxiety and depression were recorded again using the GAD-7 and PHQ-9 tests, respectively. These tests were repeated once more, three months after the end of the treatment. The thirty participants who completed the treatment were fully compliant, attended all the eight sessions and filled out both questionnaires at the pre-test, post-test and follow-up stages.

After the three-month follow-up test, all thirty participants were invited to take part in a non-directive semi-structured interview with A.E., who had had no previous contact with them, to examine what changes if any they had experienced. Eleven participants took part in these interviews which were audiotaped. Those who declined to be interviewed cited as reason the long commute from their homes in the suburbs to the location in the city centre where the interviews were taking place.

## 3. Data Analysis and Results

Formally, the basis of the study consisted of the following.

The null hypothesis: SAT intervention does not lead to any significant change in the levels of anxiety and depression as measured by GAD-7 and PHQ-9.

The alternative hypothesis: SAT intervention induces a significant change in the levels of anxiety and depression as measured by GAD-7 and PHQ-9.

The data obtained at the three evaluation stages (pre-test: Baseline; post-test: Week 12; three-month follow-up test: Week 24) during this longitudinal study to measure the effect of SAT on anxiety and depression was analyzed by ANOVA procedure with three independent variables corresponding to the measurements at pre-test, post-test and follow-up test and two dependent variables, namely, anxiety and depression. When the data is normally distributed, as was the case here (see below), ANOVA is used as a parametric test, which determines the probability *p* that the data could have occurred by random chance, meaning that the null hypothesis is true. One usually takes *p* = 0.05 as the cut-off value, below which the null hypothesis should be rejected. The smaller the value of *p* the stronger the evidence for the alternative hypothesis. Cohen’s effect size is also used to measure the effect of the intervention: an effect size of 0.2 is considered as small, 0.5 as moderate and an effect size greater than 0.8 is regarded as large. Our data was statistically reviewed using the SPSS Statistics 22.0 software (IBM, Armonk, NY, USA).

The statistics of age and scores for GAD-7 and PHQ-9 in the pre-test, post-test and three-month follow-up test are given in Table 1. One of the assumptions of the repeated measurement analysis was the normal distribution of dependent variables, and the Kolmogorov-Smirnov test was used to verify this assumption: This test did not result in statistical significance in pre-test scores for anxiety and depression. These two variables have thus normal distribution and can be used in the parametric analysis of the results.

Another assumption of variance analysis with repeated measurements is the uniformity of the variance-covariance matrix for dependent variables. This assumption was checked by Mauchly’s Test of Sphericity. Mauchly’s statistics do not result in statistical significance for either anxiety or depression. This means that the variance/covariance matrix is uniform; in other words, the distribution of variance differences in repeated combinations of both dependent variables is uniform.

To determine the process of change, and whether there is a greater change immediately after treatment, or during the follow-up period (three months after the end of the treatment), we used the Bonferroni comparison post-hoc test. The results are listed in Table 2, Table 3 and Table 4 which respectively compare pre-test scores with post-test scores, pre-test scores with follow-up scores and the post-test scores with follow-up scores.

As we can see, from Table 2 and Table 3, the differences in anxiety in pre-test and post-test and in pre-test and follow-up test, respectively, are both significant at *p* < 0.001. Cohen’s effect size, with correction factor for small sample size <50, comparing pre-test and post-test scores is 2.5 whereas comparing pre-test and follow-up test scores the effect size is 3.5. The difference between post-test and follow-up test for anxiety, as given in Table 4, is significant at *p* < 0.05 and its effect size is 0.6. This shows that the rate of change during the treatment is greater than in the time following treatment. In other words, following the period of treatment, the level of anxiety continues to drop at a lower rate.

For depression the difference between pre-test and post-test, and between pre-test and follow-up-test scores is significant at *p* < 0.001 as seen in Table 2 and Table 3 respectively. The effect size for comparing the pre-test and post-test scores is 2.3 whereas the effect size for comparing pre-test and follow-up scores is 3.1. However, Table 4 shows that the difference between post-test and follow-up test scores for depression is not significant (*p* = 0.059) and the effect size is 0.5. This shows that during the period of treatment the level of depression shows a significant drop, but in the three months following treatment it does not show a significant change.

In the semi-structured interview conducted after the three-month follow-up test, two questions were put to the participants: (i) what were the problems and reasons they took part in the intervention, and, (ii) what, if any, change they had experienced. All 11 participants who were interviewed pointed out that they had severe inter-personal problems with their family members (parents, siblings, husbands and children). They all spontaneously reported significant improvement in their inter-personal problems after being able to bond with their childhood selves; they also spontaneously reported a significant increase in their tolerance and acceptance of their family members, as well as themselves. The majority also spontaneously pointed out a corresponding increase in their social interactions (9 out of 11) and significant improvement in home-management (8 out of 11).

## 4. Discussion

Our study, albeit with a small sample size, has shown statistically significant improvement of symptoms in female sufferers of chronic depression and anxiety by the end of interrvention with a large effect size. In the three-month follow-up, we see further statistically significant improvement and effect size in anxiety but not in depression. This difference between response to anxiety and depression is perhaps linked to a trend that has been seen in the efficacy of CBT over medication for treating depression compared to anxiety. In a large meta-study, effects for some anxiety disorders such as panic disorder significantly favored CBT over medications but for depression no significant difference was found between CBT and medication [8].

Since the distinct feature of SAT is the creation of an affectional bond, our findings show that an internal passionate love for the childhood self, informed by attachment theory, can be used for psychotherapeutic intervention. It is hypothesized that this provides a healthy method for activating the reward circuitry of the brain which releases happy hormones in the body. The induced hope, incentive and energy can then facilitate and accelerate the treatment process. The question arises of how SAT can be compared with other techniques.

Apart from attachment theory, SAT has some overlapping ideas with SFT, CFT and ACT all of which have been tested on chronic conditions as indicated in the Introduction—as well as with what is now called ‘Inner Child Therapy’ in popular psychology. We describe below the similarities and differences between SAT and these techniques. Future randomised clinical trials would be able to determine the efficacy of SAT with respect to these techniques.

In SFT, limited reparenting is used and encouraged, with the therapist adopting the role of a parent to redress early maladaptive schemas and establish a secure attachment, often with the assistance of imagery and photographs. Here, there is clearly an important similarity with SAT but also a key difference: In SAT, the individual undertakes the reparenting of their childhood self themselves rather than through a therapist. The practitioners of SFT are aware that reparenting by the therapist has its pitfalls since it can potentially lead to dependency on the therapist; they state: “[L]imited reparenting does not involve the therapist actually becoming a parent regressing the patient into childlike dependency” [56].

In CFT, the individual is encouraged to show compassion to themself and others [57]. The results of a Randomised Controlled Trial on the efficacy of this technique showed increased compassion and reduced depressive symptoms in individuals recovering from psychosis [58]. CFT has also been employed as an intervention for reducing excessive self-criticism in a VR environment where the user encounters a (Freud-like) therapist avatar [59]. It has also been the basis of a pilot VR-based study to treat depression where the patients practiced expressing compassion while inhabiting a virtual adult body and then experienced receiving compassion from themselves while inhabiting a virtual generic child body [60]. SAT and CFT have therefore an overlap as well as a major difference; in SAT, the intervention starts by connecting compassionately with the childhood self of the individual rather than a generic child and, additionally, this compassion is later enhanced into an affectional bonding. A brain simulation study has considered the hypothesis that empathic concern for another person perceived as close to the self (as in SAT) might strengthen the incentive for self-directed bonding, which can lead the individual toward more compassionate states compared to the situation when compassion is expressed to a more distant person [46].

In ACT, individuals are encouraged to endure difficult situations without trying to run away from them and “move toward valued behaviour” [61,62]. This commitment in ACT resembles the vow in SAT made by the individual to support the childhood self like a good enough parent. However, in SAT the commitment for personal growth is given in the dynamic relationship between two different agents: the adult self and a childhood self that is imaginatively externalised from the individual using their childhood photos.

Finally, SAT has some parallels with various popular techniques under the rubric of inner child healing, which have emerged in the past fifty years since the notion of the inner child was introduced [63]. A common ground in these techniques lies in support for and love of the inner child, while revisiting and reprocessing the memories of traumatic childhood episodes. While there is clearly a similarity here with SAT, the key difference is that in SAT the childhood self is represented explicitly by the childhood photos of the individual who imaginatively creates a passionate affectional bonding with the child, leading to a relationship in which the adult self actively emulates the optimal parent-child interactions in real life to comfort the child.

## 5. Conclusions

Informed by attachment theory, SAT is a new psychotherpeutic and algorithmic intervention in which the patient learns to imaginatively create an affectional and passionate bond with their childhood self, which is considered to represent the emotinal world of the patient. The adult self is trained to become an attachment object for the childhood self and, to this end, self-administers a range of protocols that emulate the optimal interactions of a good enough paraent with their child when in distress. To the best of our knowledge this was the first study to evaluate the efficacy of the self-attachment technique. Despite several limitations, our results show that SAT has the potential to be used in treating resistant depression and anxiety in women, a potential that needs to be verified by randomized controlled trials. Finally, whether SAT has the potential to treat other mental illnesses such as childhood trauma or OCD needs further investigation.

## 6. Limitations

As this study was the first pilot project to evaluate the efficacy of treating depression and anxiety with SAT, no contol group was envisaged in the project; this is the primary limitation of our study. Randomized Controlled Trials would be needed in future studies to provide further, definitive evidence of the efficacy of SAT for treating depression and anxiety. Another issue is that for this pilot we used the two basic GAD-7 and GPHQ-9 questionnaries to measure anxiety and depression, while our selection criteria included chronic depression and/or anxiety, conditions which were only diagnosed in a one-hour long interview. Using Beck’s inventories for anxiety and depression (BAI and BDI, respectively) would give a better measurement for the degree of these disorders in future study subjects. We also did not have a quantitative measurment for improvement in social functoining and only had a qualitative measurement based on interviews carried out with the participants after the three-month follow-up; however only 11 participants could be interviewed. In addition, a related issue is that the selection of the participants took place only based on the level and duration of anxiety and.or depression and a proper diagnosis was not carried out. A third limitation was the selection of only female subjects between 26 and 43 years of age. Studies of SAT interventions which include both men and women in other countries are required for future work.

## Figures and Tables

**Table 1:** Statistics of Age and Anxiety and Depression Scores in Pre-test, Post-test and Follow-up test for 30 Women.

	N	Minimum	Maximum	Mean	Std. Deviation
Age	30	26.0	43.0	34.5	4.2
Pre-anxiety	30	6.0	21.0	14.2	4.1
Post-anxiety	30	0.0	18.0	4.0	3.7
Follow-up-anxiety	30	0.0	8.0	2.0	2.2
Pre-depression	30	6.0	27.0	16.4	5.6
Post-depression	30	0.0	21.0	4.0	4.4
Follow-up-depression	30	0.0	11.0	2.0	2.7

**Table 2:** Scores at Baseline and After 12 Weeks of Self-Attachment for 30 Subjects with Chronic Depression or Anxiety.

Score						
	Baseline		Week 12		Analysis	
Measure	Mean	SD	Mean	SD	Effect Size ^a^	*p* ^b^
GAD-7 (N = 30)	14.2	4.1	4.0	3.7	2.5	0.001
PHQ-9 (N = 30)	16.4	5.6	4.0	4.4	2.3	0.001

^a^ Within-group effect size (Cohen’s d with correction factor for small sample size <50); ^b^ Adjustment for multiple comparisons: Bonferroni.

**Table 3:** Scores at Baseline and the Follow-up 12 Weeks after the End of Self-Attachment Intervention.

Score						
	Baseline		Week 24		Analysis	
Measure	Mean	SD	Mean	SD	Effect Size ^a^	*p* ^b^
GAD-7 (N = 30)	14.2	4.1	2.0	2.2	3.5	0.001
PHQ-9 (N = 30)	16.4	5.6	2.0	2.7	3.1	0.001

^a^ Within-group effect size (Cohen’s d with correction factor for small sample size <50); ^b^ Adjustment for multiple comparisons: Bonferroni.

**Table 4:** Scores at Post-test and the Follow-up 12 Weeks after the End of Self-Attachment Intervention.

Score						
	Week 12		Week 24		Analysis	
	Mean	SD	Mean	SD	Effect Size ^a^	*p* ^b^
GAD-7 (N = 30)	4.0	3.7	2.0	2.2	0.6	0.001
PHQ-9 (N = 30)	4.0	4.4	2.0	2.7	0.5	0.059

^a^ Within-group effect size (Cohen’s d with correction factor for small sample size <50); ^b^ Adjustment for multiple comparisons: Bonferroni.

## Data Availability

The participants’ anonymized data can be obtained for research purposes only from the corresponding author.

## References

[1] Goldberg D., Huxley P. (1992). Common Mental Disorders: A Bio-Social Model.

[2] Vos T., Allen C., Arora M., Barber R.M., Bhutta Z.A., Brown A., Liang X., Kawashima T., Coggeshall M. (2015). Global, regional, and national incidence, prevalence, and years lived with disability for 301 acute and chronic diseases and injuries in 188 countries, 1990–2013: A systematic analysis for the Global Burden of Disease Study 2013. Lancet.

[3] Steel Z., Marnane C., Iranpour C., Chey T., Jackson J.W., Patel V., Silove D. (2014). The global prevalence of common mental disorders: A systematic review and meta-analysis 1980–2013. Int. J. Epidemiol..

[4] Patel V., Saxena S., Lund C., Thornicroft G., Baingana F., Bolton P., Chisholm D., Collins P.Y., Cooper J.L., Eaton J. (2018). The Lancet Commission on global mental health and sustainable development. Lancet.

[5] Sharp D., Morrell D. (2018). The Psychiatry of General Practice. The Scope of Epidemiological Psychiatry.

[6] Lloyd K., Jenkins R. (1995). Chronic depression and anxiety in primary care: Approaches to liaison. Adv. Psychiatr. Treat..

[7] Lyons R., Caroll D., Doherty K., Flynn M., Mason J., O’Brien D., O’Kelly F. (1992). General practice estimates of the prevalence of common chronic conditions. Ir. Med. J..

[8] Moghaddam B.R., Pauly M.C., Atkins D.C., Baldwin S.A., Stein M.B., Roy-Byrne P. (2011). Relative effects of CBT and pharmacotherapy in depression versus anxiety: Is medication somewhat better for depression, and CBT somewhat better for anxiety?. Depress. Anxiety.

[9] McHugh R.K., Whitton S.W., Peckham A.D., Welge J.A., Otto M.W. (2013). Patient preference for psychological vs. pharmacologic treatment of psychiatric disorders: A meta-analytic review. J. Clin. Psychiatry.

[10] Torpey D., Klein D. (2008). Chronic depression: Update on classification and treatment. Curr. Psychiatry Rep..

[11] American Psychiatric Association (2013). Diagnostic and Statistical Manual of Mental Disorders: DSM-5.

[12] Klerman G., Weissman M. (1994). Interpersonal Psychotherapy of Depression: A Brief, Focused, Specific Strategy.

[13] Matusiewicz A.K., Hopwood C.J., Banducci A.N., Lejuez C. (2010). The Effectiveness of Cognitive Behavioral Therapy for Personality Disorders. Psychiatr. Clin. N. Am..

[14] Van Vreeswijk M., Broersen J., Nadort M. (2012). The Wiley-Blackwell Handbook of Schema Therapy: Theory, Research, and Practice.

[15] Kahl K., Winter L., Schweiger U. (2012). The third wave of cognitive behavioural therapies: What is new and what is effective?. Curr. Opin. Psychiatry.

[16] Jakobsen J.C., Gluud C., Kongerslev M., Larsen K.A., Sørensen P., Winkel P., Lange T., Søgaard U., Simonsen E. (2014). Third-wave cognitive therapy versus mentalisation-based treatment for major depressive disorder: A randomised clinical trial. BMJ Open.

[17] Allen J., Fonagy P., Bateman A. (2008). Mentalizing in Clinical Practice.

[18] Bowlby J. (1969). Attachment and Loss: Attachment.

[19] Bowlby J. (1973). Attachment and Loss: Volume II: Separation, Anxiety and Anger.

[20] Bowlby J. (1980). Attachment and Loss: Volume III: Loss, Sadness and Depression.

[21] Cassidy J., Shaver P. (1999). Handbook of Attachment: Theory, Research, and Clinical Applications.

[22] Mikulincer M., Shaver P. (2007). Attachment in Adulthood: Structure, Dynamics, and Change.

[23] Mikulincer M., Shaver P. (2012). An Attachment Perspective on Psychopathology. World Psychiatry.

[24] Schore A.N. (2003). Affect Dysregulation and Disorders of the Self.

[25] Cozolino L. (2014). The Neuroscience of Human Relationships: Attachment and the Developing Social Brain.

[26] Courtois C., Ford J. (2012). Treatment of Complex Trauma: A Sequenced, Relationship-Based Approach.

[27] Bowlby J. (1955). The Growth of Independence in the Young Child. J. R. Soc. Health.

[28] Kirkpatrick L., Shaver P.R. (1990). Attachment theory and religion: Childhood attachments, religious beliefs, and conversion. J. Sci. Study Relig..

[29] Kirkpatrick L. (2005). Attachment, Evolution, and the Psychology of Religion.

[30] Granqvist P., Mikulincer M., Shaver P. (2010). Religion as attachment: Normative processes and individual differences. Personal. Soc. Psychol. Rev..

[31] Bonab B.G., Miner M., Proctor M. (2013). Attachment to God in Islamic spirituality. J. Muslim Ment. Health.

[32] Edalat A. (2015). Introduction to self-attachment and its neural basis. Proceedings of the 2015 International Joint Conference on Neural Networks (IJCNN).

[33] Edalat A. Self-Attachment: A Self-Administrable Intervention for Chronic Anxiety and Depression. 2017. Technical Report, Department of Computing, Imperial College London. https://www.doc.ic.ac.uk/research/technicalreports/2017/DTRS17-3.pdf.

[34] Edalat A. (2017). Self-attachment: A holistic approach to computational psychiatry. Computational Neurology and Psychiatry.

[35] Winnicott D.W. (2021). The Child, the Family, and the Outside World.

[36] Freud S. (2012). The Future of an Illusion.

[37] Bartels A., Zeki S. (2000). The neural basis of romantic love. Neuroreport.

[38] Acevedo B.P., Aron A., Fisher H.E., Brown L.L. (2012). Neural correlates of long-term intense romantic love. Soc. Cogn. Affect. Neurosci..

[39] Bartels A., Zeki S. (2004). The neural correlates of maternal and romantic love. Neuroimage.

[40] Schjødt U., Stødkilde-Jørgensen H., Geertz A.W., Roepstorff A. (2008). Rewarding prayers. Neurosci. Lett..

[41] Wise R. (2004). Dopamine, learning and motivation. Nat. Rev. Neurosci..

[42] Edalat A., Mancinelli F. (2013). Strong attractors of Hopfield neural networks to model attachment types and behavioural patterns. Proceedings of the 2013 International Joint Conference on Neural Networks (IJCNN).

[43] Edalat A. (2013). Capacity of strong attractor patterns to model behavioural and cognitive prototypes. Advances in Neural Information Processing Systems 26.

[44] Cittern D., Nolte T., Friston K., Edalat A. (2018). Intrinsic and extrinsic motivators of attachment under active inference. PLoS ONE.

[45] Numan M. (2014). Neurobiology of Social Behavior: Toward an Understanding of the Prosocial and Antisocial Brain.

[46] Cittern D., Edalat A. (2017). A neural model of empathic states in attachment-based psychotherapy. Comput. Psychiatry.

[47] Cittern D., Edalat A. (2015). Towards a neural model of bonding in self-attachment. Proceedings of the 2015 International Joint Conference on Neural Networks (IJCNN).

[48] Polydorou N., Edalat A. (2021). An interactive VR platform with emotion recognition for self-attachment intervention. EAI PHAT.

[49] Alazraki L., Ghachem A., Polydorou N., Khosmood F., Edalat A. (2021). An empathetic AI coach for self-attachment therapy. Proceedings of the 2021 IEEE Third International Conference on Cognitive Machine Intelligence (CogMI).

[50] Browne R.H. (1995). On the use of a pilot sample for sample size determination. Stat. Med..

[51] Lancaster G.A., Dodd S., Williamson P.R. (2004). Design and analysis of pilot studies: Recommendations for good practice. J. Eval. Clin. Pract..

[52] Plummer F., Manea L., Trépel D., McMillan D. (2016). Screening for anxiety disorders with the GAD-7 and GAD-2: A systematic review and diagnostic metaanalysis. Gen. Hosp. Psychiatry.

[53] Negeri Z.F., Levis B., Sun Y., He C., Krishnan A., Wu Y., Bhandari P.M., Neupane D., Brehaut E., Benedetti A. (2021). Accuracy of the Patient Health Questionnaire-9 for screening to detect major depression: Updated systematic review and individual participant data meta-analysis. BMJ.

[54] Kroenke K., Wu J., Yu Z., Bair M.J., Kean J., Stump T., Monahan P.O. (2016). The patient health questionnaire anxiety and depression scale (PHQ-ADS): Initial validation in three clinical trials. Psychosom. Med..

[55] Farah S. (2021). Jung’s Dream House and Discovering Your Own Archetypal Home. https://appliedjung.com/jungs-dream-house/.

[56] Young J., Klosko J., Weishaar M. (2006). Schema Therapy: A Practitioner’s Guide.

[57] Gilbert P. (2009). Introducing compassion-focused therapy. Adv. Psychiatr. Treat..

[58] Braehler C., Gumley A., Harper J., Wallace S., Norrie J., Gilbert P. (2013). Exploring change processes in compassion focused therapy in psychosis: Results of a feasibility randomized controlled trial. Br. J. Clin. Psychol..

[59] Falconer C.J., Slater M., Rovira A., King J.A., Gilbert P., Antley A., Brewin C.R. (2014). Embodying compassion: A virtual reality paradigm for overcoming excessive self-criticism. PLoS ONE.

[60] Falconer C.J., Rovira A., King J.A., Gilbert P., Antley A., Fearon P., Ralph N., Slater M., Brewin C.R. (2016). Embodying self-compassion within virtual reality and its effects on patients with depression. BJPsych Open.

[61] Hayes S., Strosahl K., Wilson K. (2011). Acceptance and Commitment Therapy: The Process and Practice of Mindful Change.

[62] Tjak J.G.L., Davis M.L., Morina N., Powers M.B., Smits J., Emmelkamp P. (2015). A meta-analysis of the efficacy of acceptance and commitment therapy for clinically relevant mental and physical health problems. Psychother. Psychosom..

[63] Janov A. (1970). The Primal Scream: Primal Therapy: The Cure for Neurosis.

